# Ceftriaxone-induced immune hemolytic anemia: a case report

**DOI:** 10.3389/fimmu.2025.1476563

**Published:** 2025-03-04

**Authors:** Li Wang, Yongxian Jiang, Gen Li, Liaoyun Zhang, Bo Qin, Aiyan Li

**Affiliations:** Department of Pharmacy, Sichuan Provincial Women’s and Children’s Hospital/The Affiliated Women's and Children's Hospital of Chengdu Medical College, Chengdu, China

**Keywords:** drug-induced immune hemolytic anemia, ceftriaxone, hemolysis, Coombs’ test, decreased hemoglobin

## Abstract

**Background:**

Drug-induced immune hemolytic anemia (DIIHA) is a rare but serious disease associated with various antibiotics, which is often misdiagnosed. DIIHA often leads to adverse outcomes, including organ failure and even death. Ceftriaxone is one of the most common drugs that cause DIIHA. This study reports a case of ceftriaxone-induced DIIHA.

**Case description:**

A 5-year-old patient diagnosed with community-acquired pneumonia developed a rash on the 5th day of anti-infective treatment with cefazoxime and azithromycin, followed by a rapid decline in hemoglobin levels and the presence of hemoglobin in the urine (hemoglobinuria). Laboratory analysis showed a positive Coombs’ test for the patient. The rash and hematuria subsided after cefazoxime and azithromycin were stopped and symptomatic treatment such as methylprednisolone intravenous infusion, sodium bicarbonate-alkalized urine, enoxaparin sodium anticoagulation, and antiallergic therapy. 5 days later, the child was treated with ceftriaxone for anti-infective therapy because the pneumonia was not healed. During the treatment, the patient suddenly developed itching all over the body, pale face, slightly fast breathing, vomiting, abdominal pain, and low back pain. Immediate cessation of ceftriaxone sodium infusion, continuous nasal catheter oxygen inhalation, normal saline dilatation, and meprednisolone anti-inflammatory and symptomatic treatment of cetirizine were performed. On the evening of the same day, the patient presented with symptoms of wine-colored urine. Laboratory analysis indicated severe anemia and persistent hemolysis, which was considered to autoimmune hemolytic anemia caused by ceftriaxone. After three transfusions and plasma exchange, the patient improved and was discharged from the hospital. One month later, there were no obvious abnormalities in urine analysis, blood routine analysis, reticulocyte analysis, and liver function test.

**Conclusion:**

Understanding the patient’s medical history can provide critical information for the diagnosis of DIIHA, and effective management of DIIHA includes immediate discontinuation of suspected drugs, transfusion support, plasma exchange, and symptomatic medication.

## Introduction

Drug-induced immune hemolytic anemia (DIIHA) is a serious and rare adverse reaction, which often occurs in the antibiotics cefotetan, ceftriaxone, and piperacillin/tazobactam. Ceftriaxone-induced DIIHA is characterized by a sharp decline in hemoglobin. Laboratory analysis usually determines hemolysis by measuring indicators including decreased hemoglobin, low haptoglobin, elevated lactate dehydrogenase (LDH), and hyperbilirubinemia (1). The Direct Coombs test (DAT) usually has positive results. Ceftriaxone-induced DIIHA has a high rate of organ failure, and a mortality rate of at least 30%. The clinical manifestations of ceftriaxone-induced DIIHA are usually more severe in children than in adults (1, 2). Therefore, timely identification of DIIHA cases and corresponding emergency strategies can significantly improve the prognosis. In clinical treatment, immediate discontinuation of ceftriaxone is the preferred treatment (3, 4). Since DIIHA can be difficult to diagnose, it should be promptly associated with DIIHA when a sharp drop in hemoglobin is detected. Once diagnosed, it should be recorded in the patient’s medical record to avoid recurrence (5, 6)

## Case presentation

A 5-year-old male patient with the basis of lung infection was given intravenous infusion of cefazoline and azithromycin for 5 days in another hospital before, and the child appeared with scattered rash on the fifth day. After discontinuing medication and anti-allergy therapy, his rash symptoms subsided but then had multiple episodes of soy-colored urine. Finally, he was admitted to our hospital with cough, abdominal pain, and hematuria. Combined with the examination results of other hospitals and our hospital, the infection focused on the left upper and lower lobes of the lung, partial consolidation of the left lower lobe of the lung, mycoplasma pneumoniae DNA positive. The urine analysis results are as follows: dark yellow, cloudy, occultic blood 3+ (sludge red blood cells: 2–3/HP); blood routine results showed HGB 102 g/L (normal range: 112 g/L–149 g/L). Hemagglutination index disorder showed plasma D-dimer 22.22 µg/mL (normal range: 0 µg/mL–1 µg/mL) and fibrin degradation products 47 µg/mL (normal range: 0–5 µg/mL). Blood biochemical results indicated an increase in bilirubin: indirect bilirubin 41.7 µmol/L (normal range: 0 µmol/L–14 µmol/L). Myocardial enzyme profile + troponin showed the following: CK-MB 84 U/L (normal range: 0 U/L–25 U/L), LDH 3,856 U/L (normal range: 155 U/L–345 U/L), HBDH 2,572 U/L (normal range: 72 U/L–182 U/L), and TnI 0.061 ng/mL (normal range: 0 U/L–0.04 U/L). Based on the results of laboratory analysis, considering hemoglobinuria and positive DAT, the patient was diagnosed with autoimmune hemolytic anemia. Considering that the patient had severe hemolysis, symptomatic treatment such as methylprednisolone intravenous infusion, sodium bicarbonate-alkalized urine, and enoxaparin sodium anticoagulation was used. The hemolysis symptoms of the child gradually improved.

**Figure 1:**
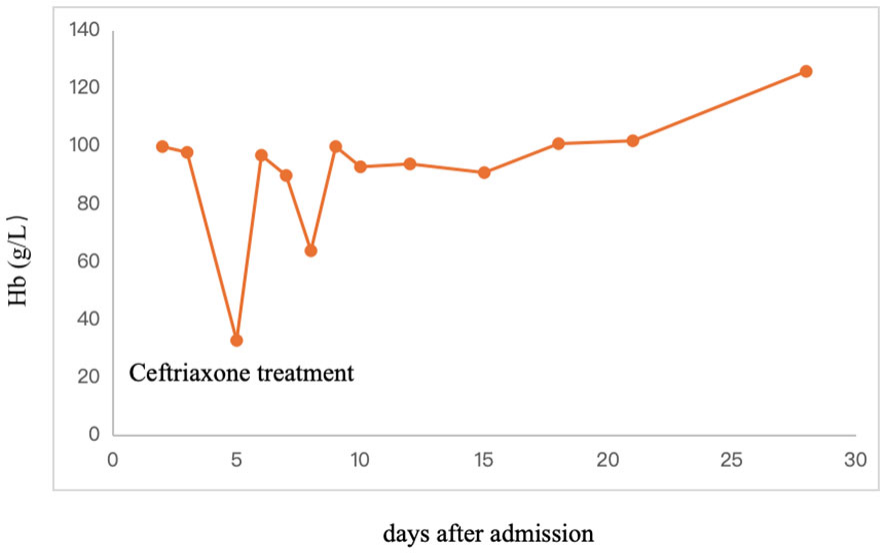
Hb of patients for different days after admission.

After 5 days, due to inflammation of the lower lobe of the patient’s left lung and partial consolidation, possibly combined with bacterial infection, ceftriaxone was added to fight infection. During the infusion of ceftriaxone, the patient suddenly developed itching all over the body, pale face, abdominal pain, low back pain, slightly fast breathing, and vomiting once. Physical examination showed the following results: T: 36.7°C, P: 105 times/min, R: 31 times/min, blood pressure 104/54 mmHg. Medication was immediately stopped, whereas continuous nasal catheter oxygen inhalation, blood pressure, ECG and blood oxygen saturation monitoring, and normal saline dilatation were performed. When meprednisolone anti-inflammatory treatment and cetirizine symptomatic treatment were used, the rashes subsided, and the complexion was normal. Laboratory analysis results showed that HGB was 33 g/L (**Figure 1**), and the myocardial enzyme profile consisted of CK-MB 72 U/L, LDH 1,467 U/L, and HBDH 1,141 U/L, indicating severe anemia.

Since severe anemia and persistent hemolysis occurred after the addition of ceftriaxone during the course of the disease, ceftriaxone-induced DIIHA was considered. The patient was subjected to three times of blood transfusion and plasma exchange (plasma replacement was performed 7 h after ceftriaxone systemic anaphylaxis), 800 ml fresh frozen plasma with AB RHD (+) was used for plasma exchange. 12 h later, the patient still had repeated hemolysis and dark-red urine appeared, and plasma replacement was performed immediately; 600 ml fresh frozen plasma was used for plasma exchange. 18 h later, due to repeated disease and severe hemolysis, the condition improved after two plasmapheresis, and the condition was stabilized, and the third plasmapheresis was performed. 850 ml of fresh frozen plasma was used for plasmapheresis; anti-coagulation therapy with methylprednisolone (40 mg, ivgtt, tid, 8 days); and enoxparin sodium (800 U, iv, st, 7 days), oral calcium (600 mg, p.o., qd), and vitamin D (400U, p.o., qd) treatment. Laboratory analysis results showed the following myocardial enzyme profile: CK-MB 14 U/L, LDH 497 U/L, HBDH 482 U/L; IBIL: 9.5 µmol/L, which led to a gradual recovery of the patient. One month later, there were no obvious abnormalities in urine analysis, blood routine analysis, reticulocyte analysis, and liver function test.

## Discussion

DIIHA is a serious and rare adverse drug reaction that can lead to adverse outcomes for patients if recognition is delayed. The disease, which is often not diagnosed in a timely manner in the clinic, affects approximately one in a million people per year (3). It has been reported that about 130 drugs may cause DIIHA, and the most common cause of drug-induced hemolytic anemia is cephalosporins (20.80%), of which ceftriaxone accounts for 37.50% (7). Ceftriaxone can kill a variety of gram-negative bacteria, gram-positive bacteria, and anaerobic bacteria and is mostly used in the treatment of sensitive infections of the lower respiratory tract, urinary tract, biliary tract, and other types (8). Ceftriaxone is widely used in adolescents and children due to its wide antibacterial spectrum and long half-life (9). Ceftriaxone-related adverse drug reactions such as anaphylactic shock, hemolysis, hematuria, and liver and kidney injury (lithiasis) continue to appear, among which acute immune hemolysis reaction is one of the more serious adverse reactions (10).

DIIHA is the result of drug-induced immune damage to red blood cells by antibodies and is classified as drug-dependent (hapten or immune complex types) and drug-independent (autoantibodies) (11, 12). The semi-anti-prototype is caused by a drug that binds firmly to the red blood cell protein or serum protein. The representative drug is penicillin. Drugs act as haptens to form holoantigens with red blood cell membranes or serum proteins, and the resulting antibodies (usually lgG type) react with the drugs attached to red blood cells, thereby damaging and destroying the drug-bound red blood cells, but not normal red blood cells.

The type of immune complex refers to the stable ternary molecular complex formed by drugs, erythrocyte membrane proteins, and anti-drug antibodies, and the immune complex non-specifically binds to the erythrocyte membrane and activates the complement, thereby destroying the red blood cells. The main antibody produced is IgG or IgM. Cefotetan, ceftriaxone, and piperacillin are its representative drugs.

Autoantibody induction type refers to the application of certain drugs, can cause changes the antigenic epitope of red blood cells, and stimulates the body to produce antibodies against own red blood cells, thus acting on the RBC to cause hemolytic anemia. Fludarabine, methyldopa, beta-lactamase inhibitors, and platinum-based chemotherapy agents are the most common agents that induce autoantibody formation. The main antibody produced is IgG (13, 14).

Ceftriaxone-induced hemolytic anemia belongs to the immune complex type and is associated with high mortality. Ceftriaxone antibodies are a necessary but not sufficient condition for ceftriaxone-induced DIIHA, and DAT is usually positive (8, 15, 16). However, DAT-negative results have also been found in ceftriaxone-induced immune hemolytic anemia, possibly because heavy hemolysis leads to a lack of complete complement/antibody-loaded red blood cells to produce a positive response (17). Therefore, a false-negative DAT result cannot rule out a diagnosis of DIIHA.

The clinical manifestations of ceftriaxone-induced hemolytic anemia are usually sudden, with sudden onset of pallor, shortness of breath, dyspnea, vomiting, headache, and lower back or abdominal pain. In severe cases, severe anemia can lead to hypovolemic shock and cardiopulmonary arrest. Laboratory manifestations include a sharp decrease in hemoglobin levels, an increase in the rete count, an increase in indirect bilirubin levels, an increase in lactate dehydrogenase levels, a peripheral blood smear consistent with hemolysis, and a positive Coombs test result and IgG autoantibodies supporting the diagnosis of DIIHA (16, 18). Treatment usually includes withdrawal of ceftriaxone, along with cardiopulmonary support and blood transfusion. Since antibodies are drug-dependent, blood transfusions are generally safe. Most importantly, the suspected drug must be discontinued immediately when DIIHA is suspected. Drug withdrawal is the most important treatment that affects the prognosis of patients. Patients with DIIHA should be admitted to an intensive care unit for optimal supportive care. In severe cases, plasma exchange has been used to treat DIIHA. Active removal of drug-induced antibodies from a patient’s serum through plasmapheresis is helpful in patients with “drug adsorption” DIIHA or severe renal failure, where the disease-causing drug is not eliminated within the normal half-life and may result in prolonged hemolysis time and increased immune stimulation (19).

Fortunately, the patient suddenly turned pale after the infusion of ceftriaxone, and the attending doctor quickly realized that this could be a drug reaction to ceftriaxone and immediately stopped using it. After ceftriaxone was stopped, the Hb level was 33 g/L. In addition, the red blood cell count decreased significantly to 0.58 × 10^12^/L, indicating serious destruction of red blood cells, severe anemia, and hemolysis symptoms, which made doctors highly suspect that ceftriaxone caused autoimmune hemolysis again. If the adverse effects of ceftriaxone are not detected in time, Hb may worsen further, so that the patient was put at significant risk. It is vital that pediatricians remain vigilant for adverse effects. Therefore, the diagnosis in this case is more likely to be ceftriaxone-induced severe DIIHA.

Dara et al. (20) reported severe drug-induced immune hemolysis due to ceftriaxone in the case of a 15-year-old patient, where testing for the drug-dependent antibody confirmed the presence of ceftriaxone-dependent antibody. The cases we report still have limitations due to the lack of an advanced serological test for ceftriaxone-dependent antibodies, which may provide more definitive evidence of the drug’s role in hemolysis. Due to the lack of detailed description from the patient’s family, the patient’s family stated that the child had no history of cephalosporin allergy. However, after systemic allergic reaction occurred in the child given ceftriaxone, under the doctor’s questioning again, the patient’s family claimed that there was a history of cephalosporin allergy. This statement directly influenced the doctor’s judgment. Therefore, the patient’s medical history plays a crucial role in the induction of autoimmune hemolysis. Although there have been many reports of autoimmune hemolysis caused by cephalosporins, in this case, due to the lack of detailed description by the patient’s family, the doctor made a wrong judgment, so that the use of ceftriaxone induced severe autoimmune hemolysis, which also alerted clinicians to ensure that the patient presented a correct medical history to avoid adverse medical outcomes.

## Conclusion

This case demonstrates that ceftriaxone-induced severe immune hemolytic anemia is a rare and severe life-threatening complication. When the patient presents with typical hemolysis, relevant laboratory tests are performed promptly. The suspect drug must be discontinued immediately to prevent serious complications and fatal consequences. In severe cases, blood transfusion and plasmapheresis may be necessary.

## Data Availability

The raw data supporting the conclusions of this article will be made available by the authors, without undue reservation.
